# Knowledge acquisition and retention following small-group case-based learning combined with sheep head animal model training in oral and maxillofacial surgery: a single-arm quasi-experimental study

**DOI:** 10.3389/fmed.2026.1866872

**Published:** 2026-07-15

**Authors:** Ahmed Ata Alfurhud

**Affiliations:** Oral and Maxillofacial Surgery and Diagnostic Sciences Department, College of Dentistry, Jouf University, Jouf, Saudi Arabia

**Keywords:** animal model, case-based learning, dental education, knowledge retention, oral and maxillofacial surgery, Simulation-based education

## Abstract

**Background:**

Developing competence in Oral and Maxillofacial Surgery (OMFS) requires effective integration of theoretical knowledge with procedural understanding. In resource-limited educational settings, opportunities for patient-based surgical training may be restricted, highlighting the need for alternative instructional strategies. This study evaluated changes in OMFS knowledge acquisition and short-term retention following small-group case-based learning combined with sheep head animal model training among dental trainees.

**Methods:**

A single-arm, pretest–posttest quasi-experimental study was conducted among fifth-year undergraduate dental students and postgraduate dental interns at a single institution. Participants completed a standardized knowledge assessment at baseline (T0), immediately following the intervention (T1), and at a 4-week follow-up (T2). The educational intervention consisted of structured small-group case-based discussions integrated with supervised hands-on training using sheep head models. Knowledge scores were analyzed using one-way repeated measures ANOVA with Greenhouse–Geisser correction and Bonferroni-adjusted *post-hoc* comparisons. Learner perceptions were assessed using a validated 14-item Likert-scale questionnaire. Effect size was reported using partial eta squared (η^2^), and internal consistency was evaluated using Cronbach’s alpha.

**Results:**

Fifty-one participants completed all assessments (response rate 67.1%). Mean knowledge scores increased significantly from baseline (50.75 ± 14.87) to immediately post-intervention (73.57 ± 16.78) and remained significantly higher at follow-up (70.24 ± 17.17). A significant effect of time was observed [*F*(1.12, 56.12) = 151.89, *p* < 0.001], with a large effect size (partial η^2^ = 0.75). *Post-hoc* analysis demonstrated significant improvements from T0 to T1 and from T0 to T2 (*p* < 0.001 for both), with a small but significant decline between T1 and T2. Learner perceptions were highly favorable, with a mean total perception score of 81.32 ± 17.24 and excellent internal consistency (Cronbach’s α = 0.943).

**Conclusion:**

Small-group case-based learning combined with sheep head animal model training was associated with substantial improvements in OMFS knowledge and meaningful retention at 4 weeks, alongside high learner satisfaction. Although objective surgical skills were not assessed, this low-cost, simulation-enhanced educational approach may support competency development in OMFS, particularly in resource-limited training environments. Future studies employing controlled designs and objective skills-based assessments are warranted.

## 1 Introduction

Competence in Oral and Maxillofacial Surgery (OMFS) is a core outcome of undergraduate and postgraduate dental education, requiring the integration of theoretical knowledge with refined technical and procedural skills (1, 2). Traditional teaching methods in OMFS have relied heavily on didactic lectures, which, although effective for knowledge dissemination, are limited in their ability to support the acquisition of complex surgical skills and clinical decision-making (3). These limitations are particularly pronounced in developing institutions, where clinical exposure may be restricted by patient availability, ethical considerations, and concerns regarding patient safety (4).

One of the major challenges in medical and dental education, especially in surgical disciplines, is equipping students with the knowledge and skills required for effective future clinical practice (5). Simulation-based education effectively bridges the gap between theoretical instruction and clinical practice, and accumulating evidence supports the use of animal tissue-based models in surgical training (6). Such models have been shown to enhance surgical skill acquisition while minimizing patient risk, leading to improvements in procedural efficiency, technical quality, and trainee confidence, with performance in some cases approaching expert levels (7). In particular, sheep head models have been associated with high learner satisfaction and provide realistic opportunities to practice manual dexterity and instrument handling, including in endoscopic procedures (8), highlighting their value as cost-effective and authentic training tools in OMFS education (9).

Although animal models are widely used across surgical disciplines to replicate realistic tissue handling and anatomy, and systematic reviews (10, 11) highlight their advantages in providing tactile feedback and risk-free practice, evidence on the use of small-group, case-based simulation combined with animal model training for OMFS knowledge acquisition and retention remains limited.

Therefore, the present study aims to evaluate the effectiveness of a curriculum integrating small-group, case-based instructional methods with practical sheep head model training. By assessing knowledge scores before, immediately after, and 4 weeks after the intervention, the study seeks to provide evidence to support OMFS curriculum development and competency-based surgical training, particularly in resource-limited educational settings.

## 2 Aims and objectives

### 2.1 Research hypothesis

The utilization of animal head models, particularly ovine (sheep) heads, in dental education is hypothesized to improve procedural knowledge acquisition and short-term retention among dental trainees.

### 2.2 Research aims

The present study aims to evaluate the effectiveness of small-group, case-based instructional methods that incorporate practical animal model training in enhancing knowledge acquisition in OMFS among undergraduate and postgraduate dental students.

### 2.3 Primary objectives

The primary objective of this study was to determine whether small-group, case-based instruction supplemented with practical training using animal models led to a statistically significant improvement in both immediate and retained knowledge of OMF surgical procedures. Knowledge acquisition was evaluated using standardized assessments administered at baseline (T0), immediately following the intervention (T1), and at delayed follow-up (T2):

(a) To assess the baseline knowledge of OMF surgical procedures among dental participants prior to the intervention (T0).
(b) To evaluate changes in knowledge immediately following the intervention (T1).
(c) To assess the retention of knowledge at a delayed 4-week follow-up (T2).
(d) To examine participants’ perceptions of the learning experience after completing the intervention.

## 3 Materials and methods

### 3.1 Study design

This study employed a single-arm, pretest–posttest quasi-experimental design to evaluate changes in knowledge acquisition and short-term retention following a small-group, case-based educational intervention supplemented with hands-on practical training using animal models. All participants received the same intervention, and no control group was included. Knowledge was assessed at three time points: baseline (T0), immediately after the intervention (T1), and at delayed follow-up 4 weeks later (T2), enabling assessment of both immediate knowledge acquisition and retention over time.

The quasi-experimental design was selected due to ethical and logistical considerations, as withholding practical training from eligible trainees was not feasible. Internal validity was strengthened through standardized instructional delivery, consistent assessment tools, and repeated within-subject measurements.

### 3.2 Participants, sampling, and setting

All 76 eligible dental trainees at the College of Dentistry, Jouf University were invited to participate, comprising fifth-year undergraduate students and postgraduate dental interns. This represented a census of the accessible population. Participation was voluntary and required written informed consent. Study activities were conducted within the Department of Oral and Maxillofacial Surgery.

Inclusion criteria were enrolment as a fifth-year undergraduate dental student or postgraduate intern, provision of informed consent, and availability to attend the intervention and all assessments. Exclusion criteria included inability to attend training or follow-up assessments, or withdrawal of consent at any stage.

Sample size estimation was performed a priori using G*Power 3.1 for paired-sample comparisons, assuming a medium effect size (Cohen’s *d* = 0.5), two-tailed α = 0.05, and power of 0.80, yielding a minimum required sample of 34 participants (12). This was considered a conservative estimate for within-subject repeated-measures analysis.

### 3.3 Educational intervention and data collection

Following baseline assessment (T0), participants attended a 1-day structured educational workshop that integrated small-group case-based learning with supervised hands-on surgical training using sheep head models. The workshop was designed to reinforce oral and maxillofacial surgical knowledge and procedural understanding through active discussion, demonstration, and guided practice.

Participants were divided into small groups of five to six trainees to facilitate interaction, discussion, and individual feedback. Each group was supervised by faculty facilitators from the Oral and Maxillofacial Surgery and Diagnostic Sciences Department. The facilitators were responsible for guiding case-based discussions, demonstrating the procedural steps, supervising hands-on practice, correcting technique where necessary, and ensuring that participants followed standard safety and specimen-handling procedures. The same teaching faculty delivered all sessions using a standardized instructional format to minimize variation in teaching delivery.

The case-based learning component focused on common oral and maxillofacial surgical scenarios and emphasized clinical decision-making, anatomical considerations, surgical indications, procedural planning, complication avoidance, and post-operative management. This was followed by hands-on training using sheep head specimens. The surgical procedures demonstrated and practiced included lower anterior tooth extraction, lower posterior tooth extraction, incision and wound design, mucoperiosteal flap reflection, identification and preservation of relevant anatomical structures, flap repositioning and suturing, alveoloplasty and bone recontouring, marginal mandibulectomy, and mandibular block graft harvesting. These procedures were selected to expose participants to a range of basic and intermediate oral surgical principles, including tissue handling, instrument use, flap management, bone removal, and wound closure.

All participants received identical exposure to the educational content, procedural demonstrations, and supervised hands-on practice. Each participant was provided with one sheep head model to ensure standardized access to the training material and individual opportunity for practice. Knowledge retention was evaluated 4 weeks later (T2). Knowledge was assessed using a standardized instrument developed for this study, consisting of 25 short-answer and true/false items covering core oral and maxillofacial surgical knowledge and procedural understanding. Each item was weighted equally, producing a total possible score of 100. All assessments were anonymized using unique participant codes to enable linkage of repeated measures while preserving confidentiality.

**FIGURE 1:**
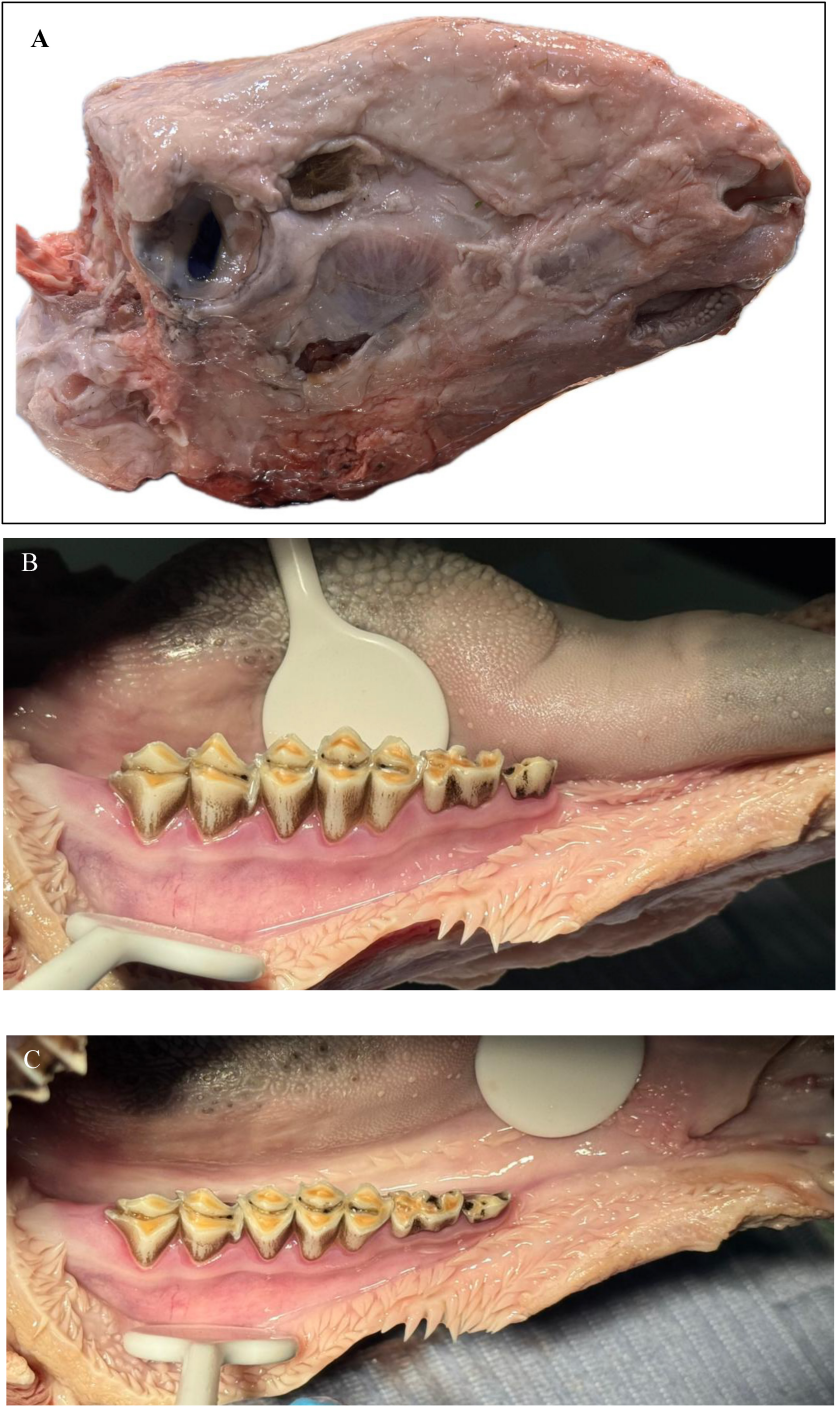
**(A)** Animal model used for surgical training. **(B,C)** Intra-operative views of the sheep head demonstrating the buccal mucosa and posterior dentition.

The assessors were not formally blinded to the assessment time point. However, participant identity was anonymized using unique codes, and scoring was conducted using a standardized answer key to minimize scoring variation. The absence of formal assessor blinding was acknowledged as a methodological limitation. Representative views of the sheep head model used during the intervention are shown in Figure 1.

### 3.4 Instrument validation

The knowledge assessment instrument consisted of 25 items covering core oral and maxillofacial surgical knowledge and procedural understanding. The instrument included five short-answer questions and 20 true/false items. All items were scored dichotomously, whereby a mark was awarded only for a correct answer, and partial marks were not awarded for the short-answer questions. The total raw score was calculated by summing all correct responses and was subsequently converted into a percentage score ranging from 0 to 100.

The same knowledge assessment instrument was administered at all three time points, namely baseline (T0), immediately after the intervention (T1), and 4-week follow-up (T2), to allow direct within-participant comparison of knowledge scores over time. The item order was not changed across the three assessments.

Content validity of the knowledge assessment instrument was evaluated by a multidisciplinary panel of six experts in oral and maxillofacial surgery, epidemiology, biostatistics, and health professions education, following established guidelines for instrument validation (13). Items were rated for relevance using a 4-point Likert scale (1 = not relevant, 4 = highly relevant), and the Item-Level Content Validity Index (I-CVI) and Scale-Level Content Validity Index (S-CVI/UA and S-CVI/AVE) were calculated. Items not meeting established thresholds were revised or removed based on expert feedback.

Face validity was assessed among 10 dental trainees who evaluated item clarity and comprehensibility using a 4-point Likert scale (1 = not clear/comprehensible; 4 = very clear/comprehensible), in accordance with recommended face validity procedures (14). Item-Level Face Validity Index (I-FVI) and scale-level indices (S-FVI/UA and S-FVI/AVE) were computed, and minor linguistic refinements were made accordingly. The face validity assessment also served as a pilot assessment of item clarity, comprehensibility, and response process prior to the main study.

The validation process focused on content validity and face validity. Internal consistency reliability of the knowledge assessment instrument was not assessed and is acknowledged as a methodological limitation.

### 3.5 Outcome measures

The primary outcome was change in knowledge scores across time (T0, T1, T2). Secondary outcomes included participants’ perceptions of the educational intervention, as well as the internal consistency reliability of the perception questionnaire.

The study outcome was limited to knowledge acquisition and procedural understanding. Objective surgical skill performance was not assessed at T0, T1, or T2 because no validated procedural skills checklist, global rating scale, or objective structured assessment was incorporated into the study protocol. Therefore, although participants practiced instrument handling, flap reflection, tissue closure, suturing, bone contouring, and graft harvesting during the workshop, these skills were not formally scored as study outcomes.

### 3.6 Statistical analysis

Primary analyses followed a per-protocol, complete-case approach, including only participants with valid data at all three assessment time points (T0, T1, and T2). Descriptive statistics were used to summarize participant characteristics and outcome measures. Normality of continuous variables was assessed using the Shapiro-Wilk test.

Changes in knowledge scores across time were analyzed using one-way repeated measures analysis of variance (ANOVA). The assumption of sphericity was evaluated using Mauchly’s test, and Greenhouse–Geisser correction was applied where sphericity was violated. *Post-hoc* pairwise comparisons were conducted with Bonferroni adjustment. Effect size was reported using partial eta squared (η^2^).

Additional mixed repeated-measures ANOVA analyses were conducted to examine whether changes in knowledge scores over time differed according to academic level and gender, which were entered separately as between-subjects factors.

Internal consistency reliability of the perception questionnaire was assessed using Cronbach’s alpha. Perception outcomes were analyzed descriptively using measures of central tendency and dispersion. All statistical tests were two-tailed, with statistical significance set at *p* < 0.05. Analyses were performed using IBM SPSS Statistics (version 31) (15).

### 3.7 Ethical considerations

Ethical approval was obtained from the Scientific Committee at the College of Dentistry, Jouf University, and the Research Ethics Committee of Jouf University (ID: HAPO-13-S-001). This study involved human participants, namely fifth-year undergraduate dental students and postgraduate dental interns, who participated in an educational intervention and completed knowledge and perception assessments. Written informed consent was obtained from all participants prior to enrolment, and participation was voluntary. Participants were informed that they could withdraw from the study at any stage without academic penalty.

Although no live animals were used, the ethical approval also covered the sourcing, handling, use, and disposal of sheep head specimens for educational purposes. All animal specimens were obtained from appropriate sources and were used solely for simulation-based surgical training. All participant data were anonymized using unique codes and securely stored in accordance with institutional data protection policies.

## 4 Results

### 4.1 Validation of the knowledge assessment instrument

#### 4.1.1 Content validity

Content validity of the knowledge assessment instrument was evaluated by a panel of six experts. All 25 items achieved an Item-Level Content Validity Index (I-CVI) of 1.00, indicating that all experts rated each item as either relevant or highly relevant (Table 1). Universal agreement was observed for all items (UA = 1). At the scale level, both the Scale-Level Content Validity Index using the Universal Agreement method (S-CVI/UA) and the Scale-Level Content Validity Index using the Average method (S-CVI/AVE) were 1.00, demonstrating excellent content validity of the instrument.

**TABLE 1:** Item-level and scale-level content validity index (CVI) for the knowledge assessment instrument.

Item	Number of experts rating 3 or 4	I-CVI	UA score
Item 1	6	1.00	1
Item 2	6	1.00	1
Item 3	6	1.00	1
Item 4	6	1.00	1
Item 5	6	1.00	1
Item 6	6	1.00	1
Item 7	6	1.00	1
Item 8	6	1.00	1
Item 9	6	1.00	1
Item 10	6	1.00	1
Item 11	6	1.00	1
Item 12	6	1.00	1
Item 13	6	1.00	1
Item 14	6	1.00	1
Item 15	6	1.00	1
Item 16	6	1.00	1
Item 17	6	1.00	1
Item 18	6	1.00	1
Item 19	6	1.00	1
Item 20	6	1.00	1
Item 21	6	1.00	1
Item 22	6	1.00	1
Item 23	6	1.00	1
Item 24	6	1.00	1
Item 25	6	1.00	1

Mean I-CVI = 1.00; S-CVI/UA = 1.00; S-CVI/AVE = 1.00. I-CVI represents the proportion of experts rating an item as relevant (3 or 4). UA denotes universal agreement.

#### 4.1.2 Face validity

Face validity was assessed among 10 dental trainees. Item-Level Face Validity Index (I-FVI) values ranged from 0.90 to 1.00, indicating a very high level of agreement regarding item clarity and comprehensibility (Table 2). Eleven items achieved universal agreement (UA = 1), while the remaining items demonstrated high agreement (I-FVI = 0.90). The mean I-FVI across all items was 0.94. At the scale level, the S-FVI/UA was 0.44, reflecting the conservative nature of universal agreement, whereas the S-FVI/AVE was 0.94, confirming excellent overall face validity of the instrument.

**TABLE 2:** Item-level and scale-level face validity index (FVI) for the knowledge assessment instrument.

Item	Respondents in agreement	I-FVI	UA
Item 1	9	0.9	0
Item 2	10	1.0	1
Item 3	9	0.9	0
Item 4	9	0.9	0
Item 5	9	0.9	0
Item 6	10	1.0	1
Item 7	9	0.9	0
Item 8	9	0.9	0
Item 9	9	0.9	0
Item 10	9	0.9	0
Item 11	10	1.0	1
Item 12	10	1.0	1
Item 13	10	1.0	1
Item 14	10	1.0	1
Item 15	10	1.0	1
Item 16	9	0.9	0
Item 17	9	0.9	0
Item 18	9	0.9	0
Item 19	10	1.0	1
Item 20	10	1.0	1
Item 21	10	1.0	1
Item 22	9	0.9	0
Item 23	9	0.9	0
Item 24	9	0.9	0
Item 25	10	1.0	1

Mean I-FVI = 0.94; S-FVI/UA = 0.44; S-FVI/AVE = 0.94. UA = 1 indicates full agreement among all respondents. I-FVI values ≥ 0.9 represent a very high level of agreement among reviewers. S-FVI/UA denotes the proportion of items with UA = 1, while S-FVI/AVE represents the average I-FVI across all items.

### 4.2 Participant characteristics

Of the 76 eligible dental trainees invited to participate, 51 completed all required assessments and were included in the final analysis, yielding an overall response and completion rate of 67.1%. The analyzed sample comprised 28 fifth-year undergraduate students (54.9%) and 23 postgraduate interns (45.1%). Majority of participants were female (*n* = 35, 68.6%), while males accounted for 31.4% (*n* = 16).

Baseline knowledge scores (T0) differed significantly by academic level, with postgraduate interns demonstrating higher mean scores than fifth-year undergraduates (59.83 ± 16.51 vs. 43.29 ± 7.70, *p* < 0.001). A statistically significant difference in baseline knowledge scores was also observed by gender, with male participants scoring higher than female participants (58.00 ± 18.42 vs. 47.43 ± 11.81, *p* = 0.047) (Table 3).

**TABLE 3:** Baseline knowledge scores (T0) by participant characteristics (*n* = 51).

Characteristics	n (%)	Mean ± SD	*P*
Academic level			
Fifth-year undergraduate	28 (54.9)	43.29 ± 7.70	< 0.001
Postgraduate intern	23 (45.1)	59.83 ± 16.51	
Gender			
Male	16 (31.4)	58.00 ± 18.42	0.047
Female	35 (68.6)	47.43 ± 11.81	

Values are presented as mean ± standard deviation for baseline knowledge scores (T0). *P*-values were derived from independent samples *t*-tests comparing academic level and gender groups.

### 4.3 Knowledge scores across time

**FIGURE 2:**
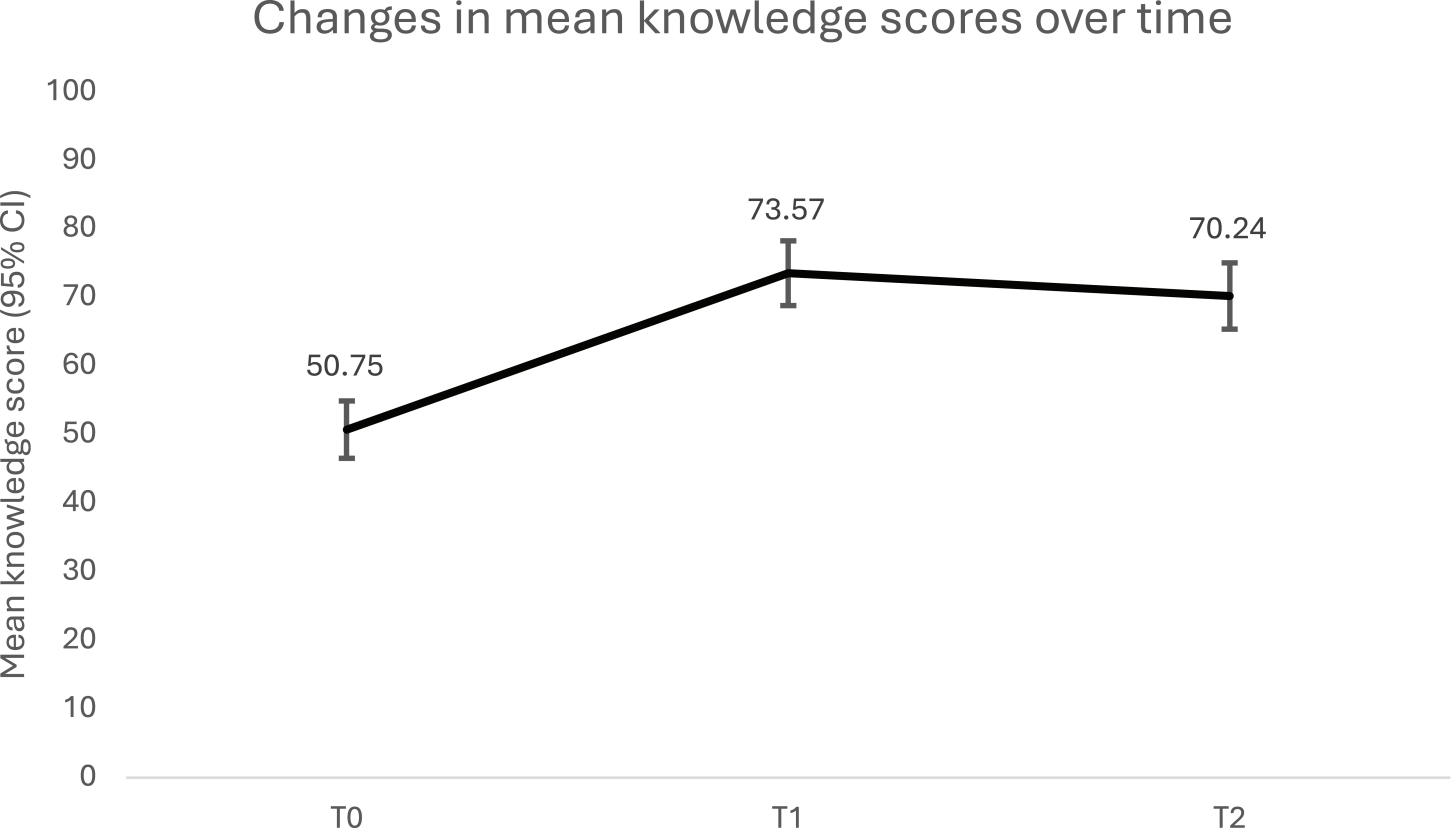
Changes in mean knowledge scores over time following the training intervention. Mean knowledge scores are presented at baseline (T0), immediately post-intervention (T1), and at 4-week follow-up (T2). Error bars represent 95% confidence intervals.

Mean knowledge scores increased following the intervention. At baseline (T0), the mean knowledge score was 50.75 ± 14.87 (range 24.00–88.00). Immediately after the intervention (T1), the mean score increased to 73.57 ± 16.78 (range 40.00–100.00). At 4-week follow-up (T2), knowledge scores remained elevated at 70.24 ± 17.17 (range 32.00–100.00), although a slight decline from T1 was observed (Table 4 and Figure 2).

**TABLE 4:** Knowledge scores at baseline, immediate post-intervention, and follow-up.

Time point	Mean ± SD	Min–max
T0 (baseline)	50.75 ± 14.87	24.00–88.00
T1 (immediate post-intervention)	73.57 ± 16.78	40.00–100.00
T2 (4-week follow-up)	70.24 ± 17.17	32.00–100.00

#### 4.3.1 Changes in knowledge across time

A one-way within-subject repeated measures ANOVA was conducted to examine changes in knowledge scores across the three time points. Mauchly’s test indicated that the assumption of sphericity was violated (*W* = 0.218, *p* < 0.001); therefore, Greenhouse–Geisser correction was applied.

There was a statistically significant effect of time on knowledge scores, *F*(1.12, 56.12) = 151.89, *p* < 0.001, with a large effect size (partial η^2^ = 0.75). Bonferroni-adjusted pairwise comparisons demonstrated significant increases in knowledge from T0 to T1 (mean difference = 22.82, 95% CI 18.83–26.82, *p* < 0.001) and from T0 to T2 (mean difference = 19.49, 95% CI 15.10–23.88, *p* < 0.001). A small but statistically significant decline was observed between T2 and T1 (mean difference = −3.33, 95% CI −4.60 to −2.07, *p* < 0.001), indicating partial decay of knowledge while remaining above baseline levels (Table 5).

**TABLE 5:** Bonferroni-adjusted pairwise comparisons of knowledge scores between time points.

Comparison	Mean difference	95% CI	*P*
T1 vs. T0	22.82	18.83–26.82	<0.001
T2 vs.T0	19.49	15.10–23.88	<0.001
T2 vs. T1	−3.33	−4.60, −2.07	<0.001

#### 4.3.2 Changes in knowledge across time by academic level and gender

Additional mixed repeated-measures ANOVA analyses were conducted to examine whether changes in knowledge scores over time differed according to academic level and gender. In the model including academic level as a between-subjects factor, the effect of time remained statistically significant after Greenhouse–Geisser correction, *F*(1.127, 55.237) = 150.659, *p* < 0.001. A significant main effect of academic level was observed, *F*(1, 49) = 11.444, *p* = 0.001, indicating that postgraduate interns had higher overall knowledge scores than fifth-year students across the assessment time points. However, the time × academic level interaction was not statistically significant, *F*(1.127, 55.237) = 2.214, *p* = 0.140, suggesting that the pattern of knowledge improvement over time did not differ significantly between postgraduate interns and fifth-year students.

**FIGURE 3:**
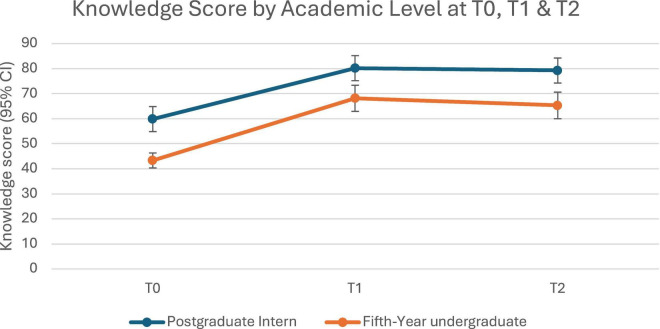
Changes in mean knowledge scores over time by academic level. Mean knowledge scores are presented for postgraduate interns and fifth-year undergraduate students at baseline (T0), immediately post-intervention (T1), and 4-week follow-up (T2). Error bars represent 95% confidence intervals. Both groups showed increased knowledge scores following the intervention, with scores remaining above baseline at 4-week follow-up.

**FIGURE 4:**
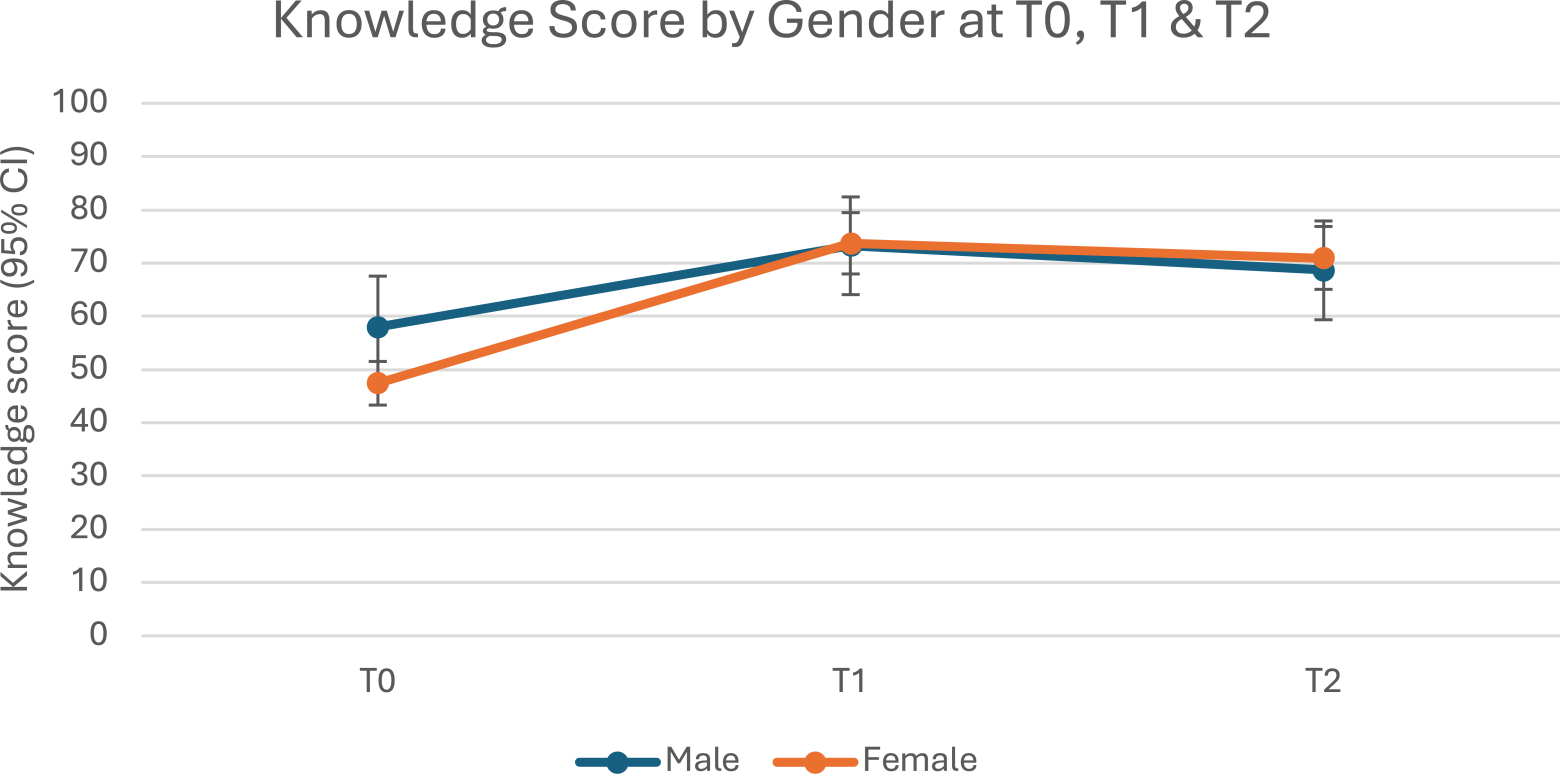
Changes in mean knowledge scores over time by gender. Mean knowledge scores are presented for male and female participants at baseline (T0), immediately post-intervention (T1), and 4-week follow-up (T2). Error bars represent 95% confidence intervals. Male participants had higher baseline scores, but post-intervention and follow-up scores were comparable between genders, indicating greater relative improvement among female participants.

In the model including gender as a between-subjects factor, the effect of time remained statistically significant after Greenhouse–Geisser correction, *F*(1.155, 56.605) = 131.028, *p* < 0.001. The main effect of gender was not statistically significant, *F*(1, 49) = 0.313, *p* = 0.579. However, a statistically significant time × gender interaction was observed, *F*(1.155, 56.605) = 12.992, *p* < 0.001. At baseline, male participants had significantly higher knowledge scores than female participants. However, no significant gender differences were observed immediately post-intervention or at 4-week follow-up. This suggests that female participants demonstrated greater relative improvement from baseline, resulting in comparable post-intervention and follow-up knowledge scores between genders (Table 6). The patterns of knowledge scores over time by academic level and gender are shown in Figures 3, 4, respectively.

**TABLE 6:** Knowledge scores across time by academic level and gender.

Subgroup	T0, mean ± SD	T1, mean ± SD	T2, mean ± SD
Academic level			
Postgraduate intern	59.83 ± 16.51	80.17 ± 18.27	76.26 ± 19.26
Fifth-year undergraduate	43.29 ± 7.70	68.14 ± 13.49	65.29 ± 13.68
Gender			
Male	58.00 ± 18.42	73.25 ± 17.69	68.63 ± 17.91
Female	47.43 ± 11.81	73.71 ± 16.61	70.97 ± 17.03

Main effect of subgroup: Academic Level–*F*(1, 49) = 11.444, *p* = 0.001; Gender–*F*(1, 49) = 0.313, *p* = 0.579. Time × subgroup interaction: Academic Level–*F*(1.127, 55.237) = 2.214, *p* = 0.140; Gender–*F*(1.155, 56.605) = 12.992, *p* < 0.001.

### 4.4 Perception of the training intervention

Responses from 44 participants were included in the perception analysis. Overall perceptions of the training intervention were favorable. The mean total perception score was 81.32 ± 17.24. Participants rated the overall appropriateness of the course in meeting their learning expectations and training needs highly (mean ± SD = 6.64 ± 1.01). Overall satisfaction with the hands-on training course using sheep heads as a surgical teaching model was similarly high (mean ± SD = 6.70 ± 0.95). Descriptive statistics for perception outcomes are presented in Table 7.

**TABLE 7:** Participants’ perceptions of the training intervention (*n* = 44).

Measure	Mean ± SD	Min–max
Total perception score (Q1–Q14)	81.32 ± 17.24	35–98
Overall course appropriateness (Q15)	6.64 ± 1.01	3–7
Overall satisfaction with training (Q16)	6.70 ± 0.95	3–7

**FIGURE 5:**
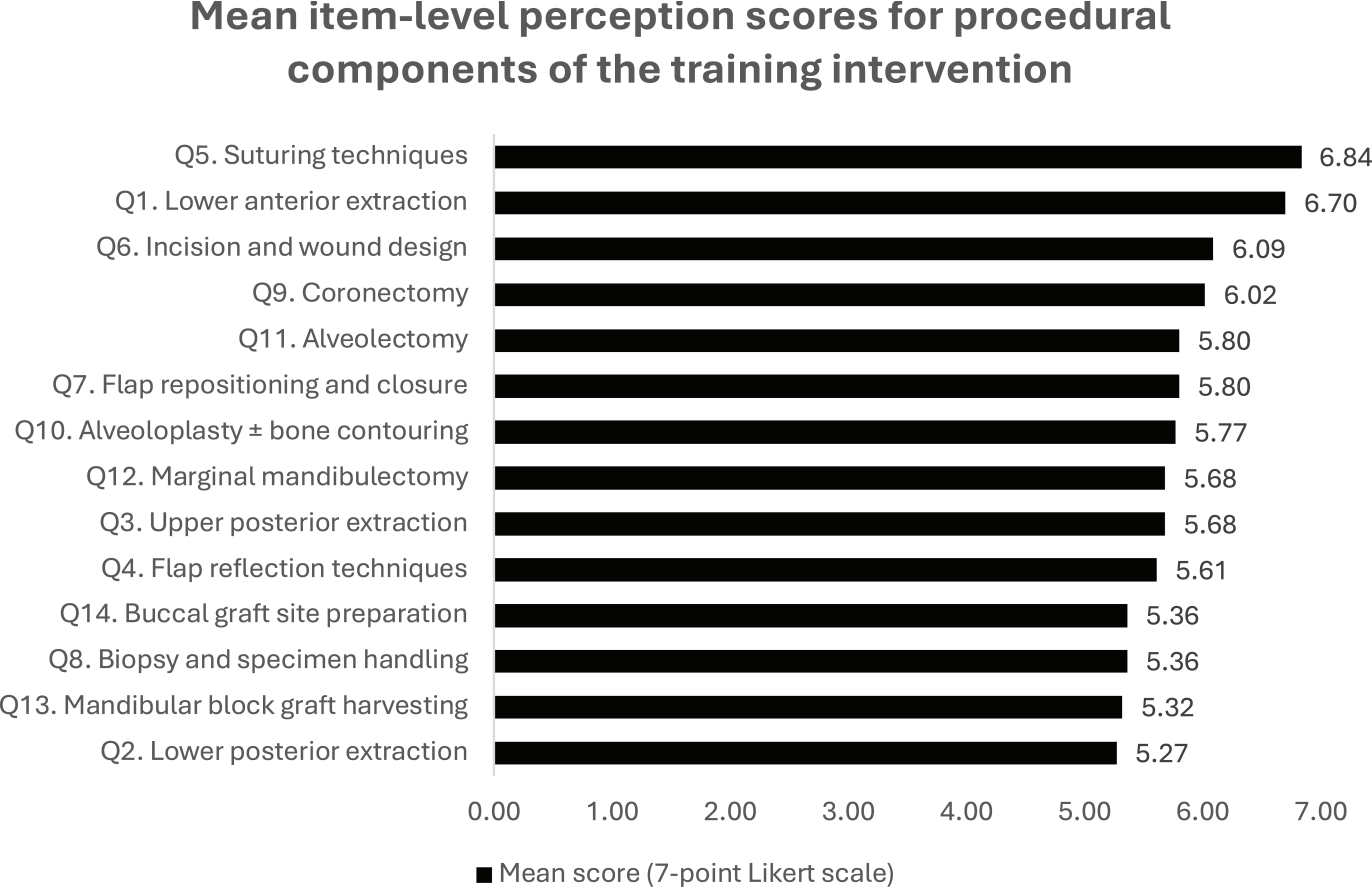
Mean item-level perception scores following the training intervention. Scores were rated on a 7-point Likert scale, with higher values indicating more favorable perceptions.

**FIGURE 6:**
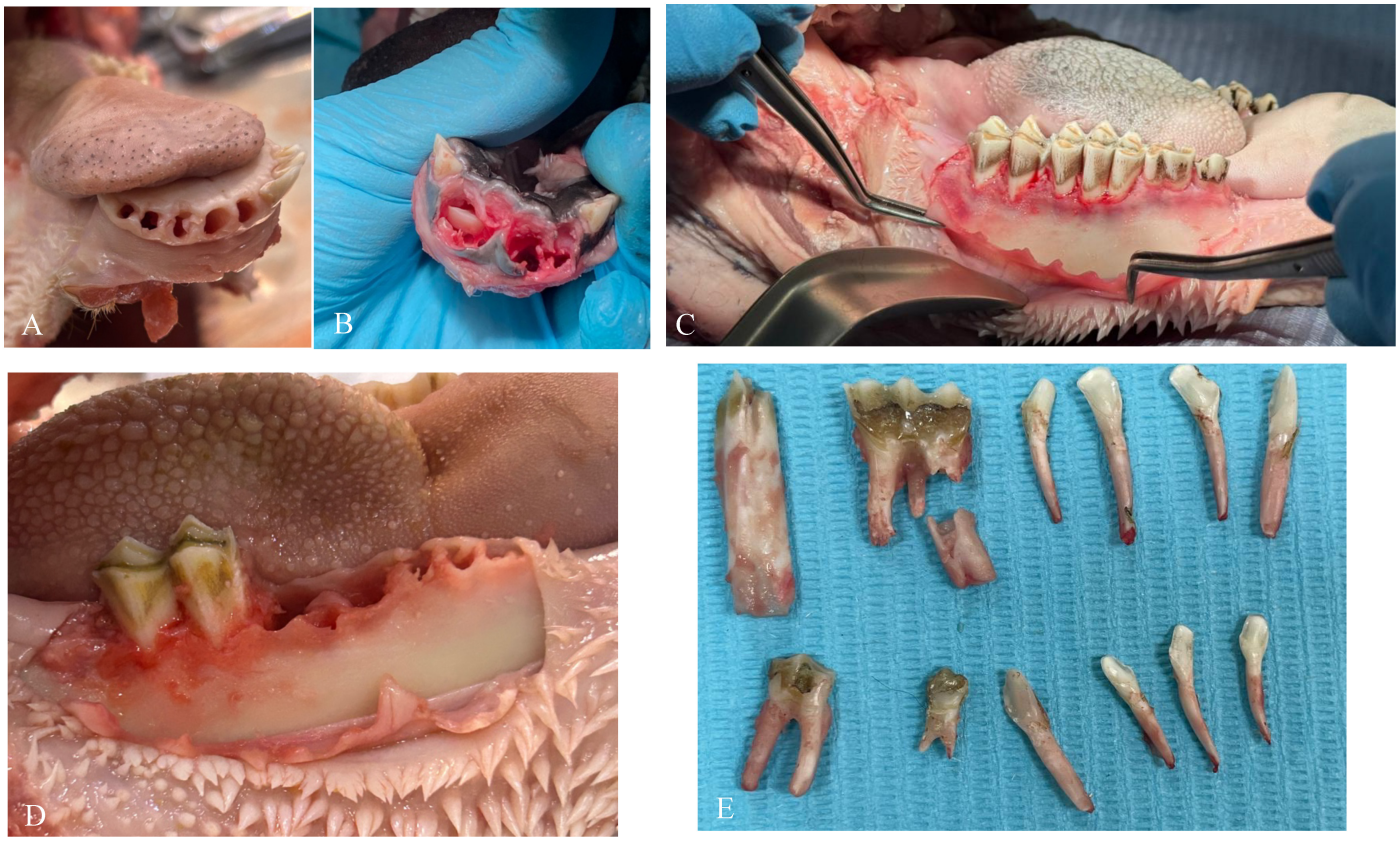
(A) Extraction sockets of the lower anterior teeth. **(B)** Impacted lower anterior tooth visualized following extraction of the erupted predecessor. **(C)** Reflection of a trapezoidal flap. **(D)** Socket following surgical extraction of lower posterior teeth. **(E)** Collection of extracted teeth.

**FIGURE 7:**
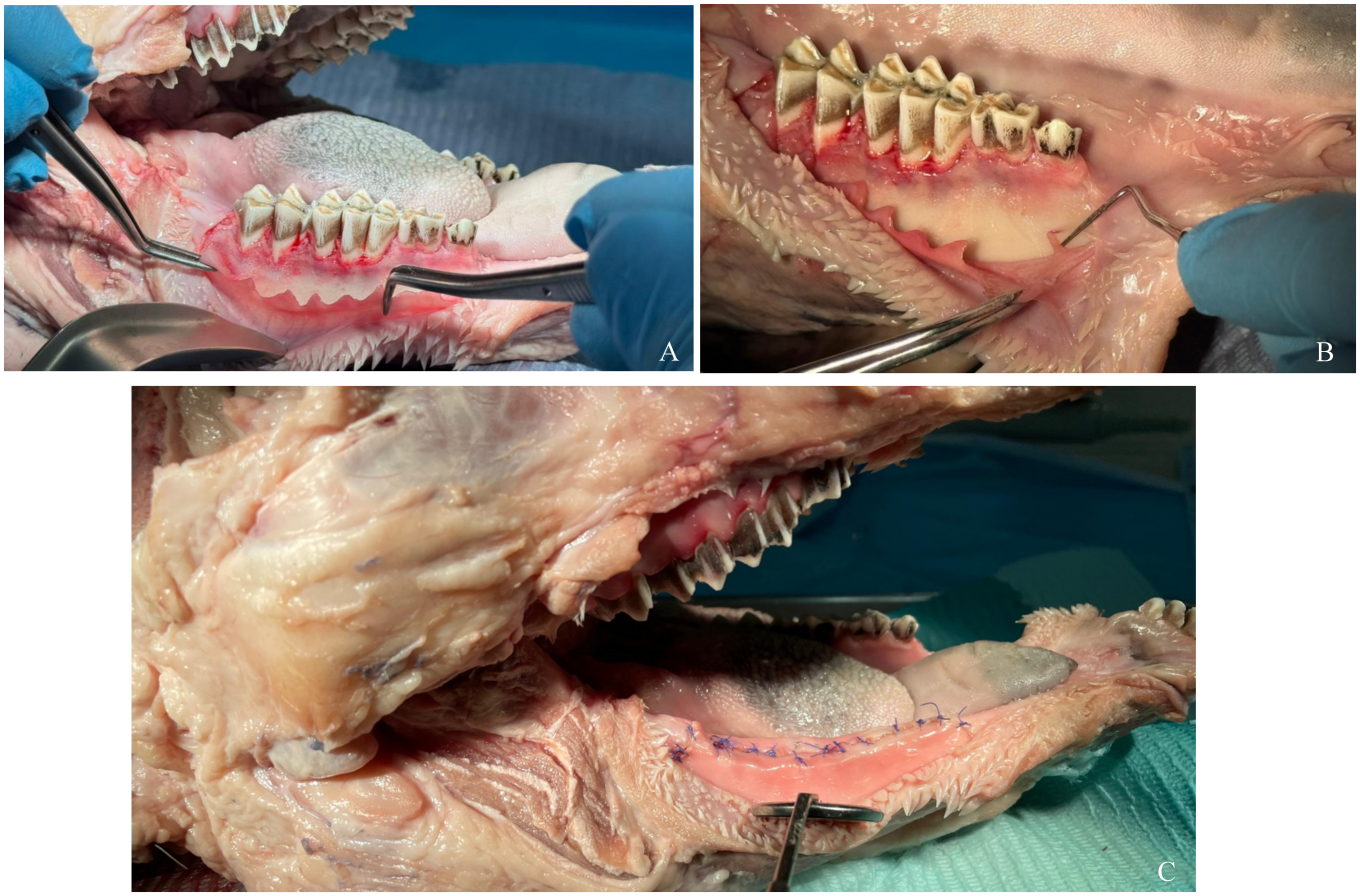
Surgical flap management and closure using the sheep head model. **(A)** Reflection of a trapezoidal mucoperiosteal flap to expose the mandibular alveolar region. **(B)** Identification and preservation of the mental nerve during flap elevation. **(C)** Flap repositioning and suturing following completion of posterior tooth extraction.

**FIGURE 8:**
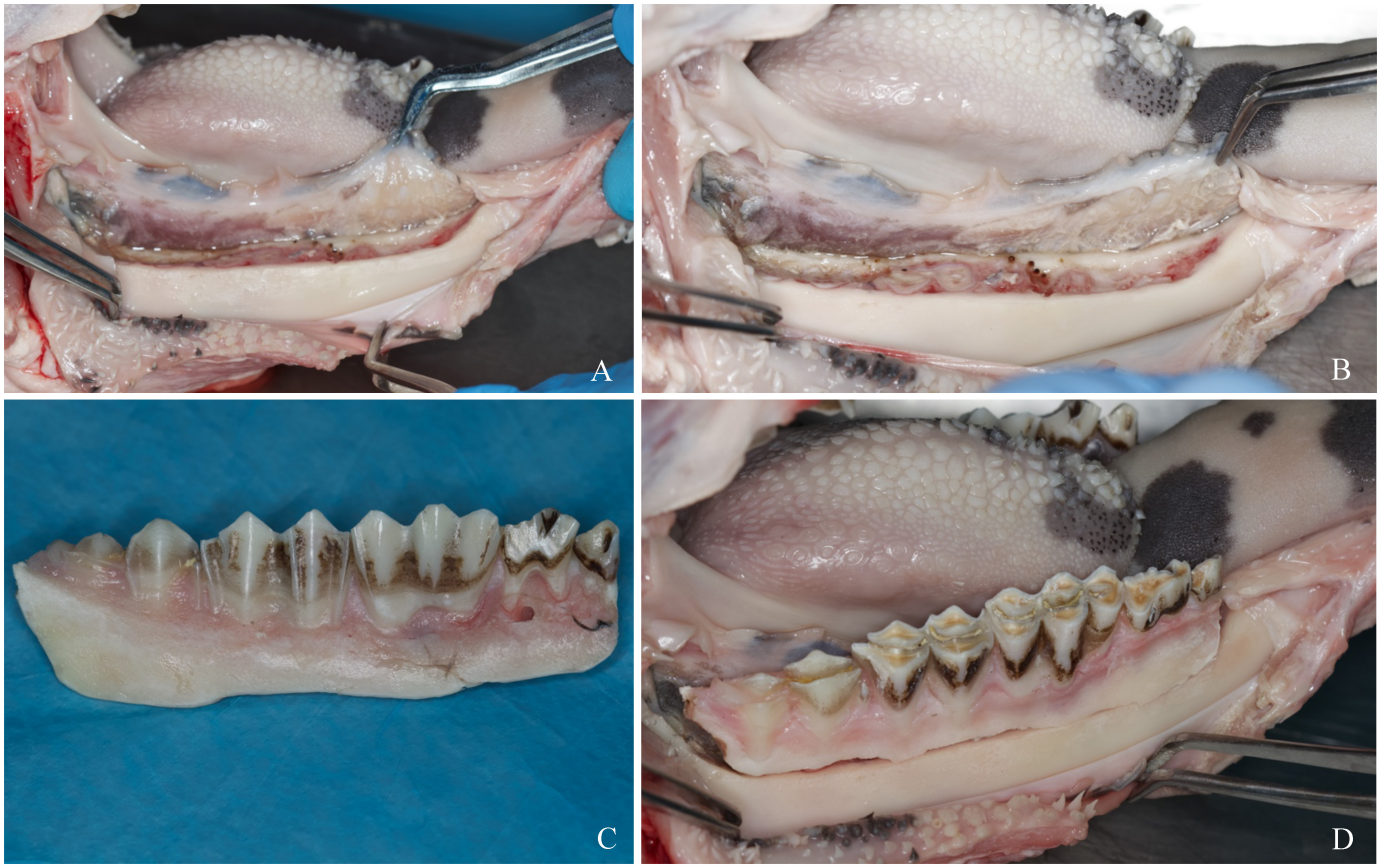
Surgical sequence for marginal mandibulectomy and alveolar bone recontouring using the sheep head model. **(A,B)** Mandibular alveolar segment following marginal bone removal and recontouring. **(C)** Resected alveolar bone segment. **(D)** Final recontoured mandibular alveolar ridge after completion of the procedure.

**FIGURE 9:**
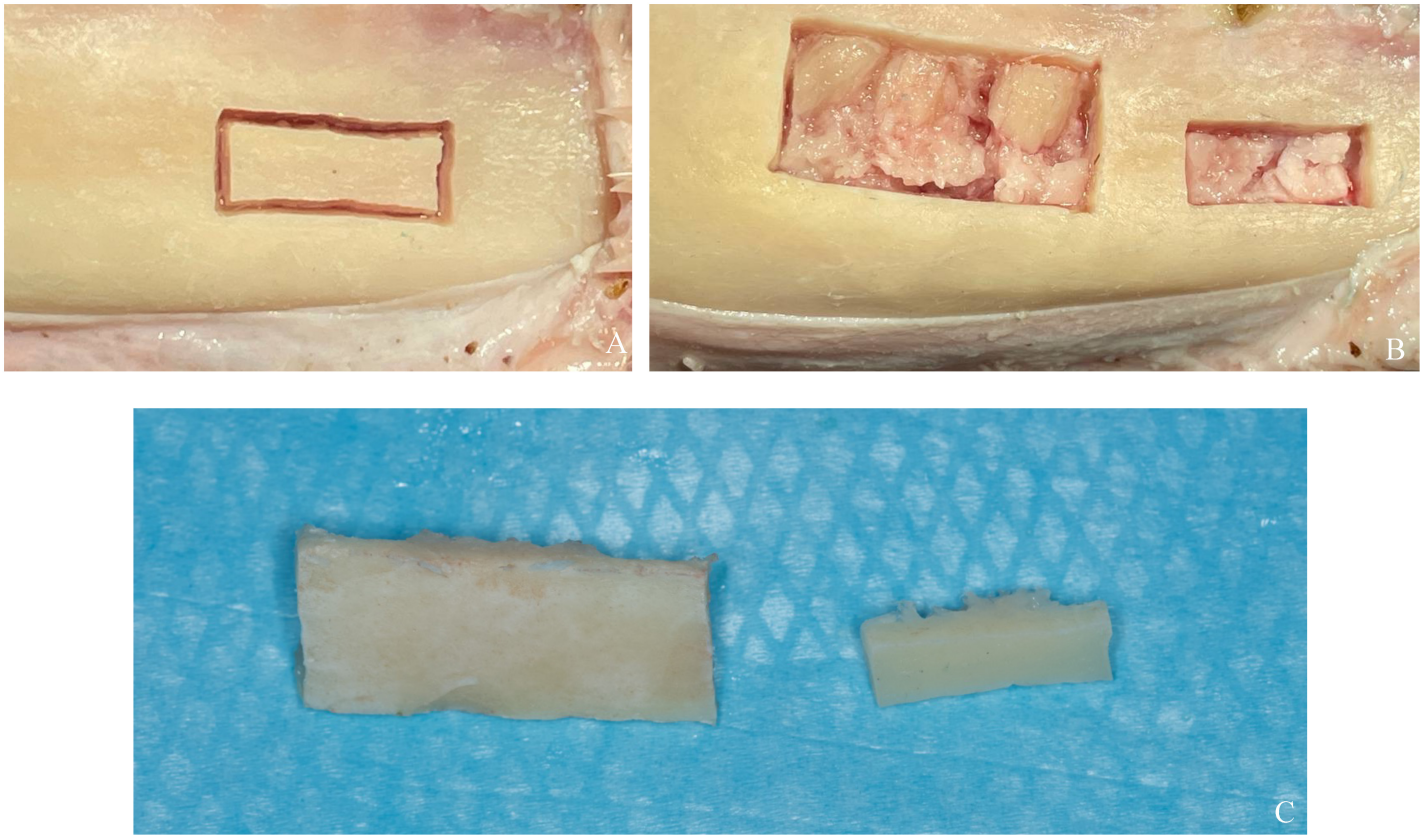
Surgical sequence for mandibular block graft harvesting using the sheep head model. **(A)** Marking and outlining of the rectangular bony window on the mandibular surface. **(B)** Removal of the cortical bone window and exposure of the underlying bone graft segment. **(C)** Harvested mandibular cortical bone blocks following completion of the grafting procedure.

Item-level analysis demonstrated consistently favorable ratings across all procedural components of the training (Figure 5). The procedural components of the intervention encompassed a broad spectrum of OMFS skills. These included lower anterior tooth extraction (Figures 6A,B) and lower posterior tooth extraction (Figure 6D), incision and wound design, and flap reflection techniques (Figures 6C, 7A,B). Participants were additionally trained in flap repositioning and suturing techniques (Figure 7C), as well as more advanced procedures such as marginal mandibulectomy with alveoloplasty and bone contouring (Figure 8). Mandibular block graft harvesting was also demonstrated and practiced as part of the intervention (Figure 9).

Mean scores for individual items ranged from 5.27 to 6.84 on a 7-point Likert scale. The highest-rated items were related to suturing techniques and lower anterior tooth extraction, while comparatively lower, though still favorable, ratings were observed for mandibular graft harvesting and lower posterior tooth extraction. No item demonstrated a mean score below the mid-point of the scale, indicating uniformly positive perceptions across all assessed procedural domains. The internal consistency of the perception scale (items Q1–Q14) was excellent (Cronbach’s α = 0.943), indicating high reliability of the instrument.

## 5 Discussion

The present study demonstrated a substantial and statistically significant improvement in OMFS knowledge following the implementation of a small-group, case-based learning intervention supplemented with hands-on animal model training. Mean knowledge scores increased markedly from T0 to T1, with scores remaining significantly higher than baseline at the 4-week follow-up (T2), despite a modest decline from immediate post-training levels. The observed effect of time on knowledge acquisition was large, suggesting that the observed improvement in learning outcomes was educationally meaningful rather than marginal.

Integrating structured case-based discussions with animal tissue-based experiential learning was associated with improved immediate knowledge acquisition and short-term knowledge retention in OMFS education. The sustained improvement observed at delayed follow-up suggests learning beyond immediate recall, which is essential for safe surgical decision-making and clinical competence, particularly among less experienced trainees (16). These benefits may be explained by the combination of small-group, case-based learning and hands-on training with sheep head models, which supports active engagement, clinical reasoning, integration of cognitive and psychomotor skills, and deeper understanding of complex surgical concepts in a low-risk, low-cost environment (8, 17, 18). These findings suggest that the integrated intervention may be a useful adjunct to conventional teaching, although direct comparison with lecture-based approaches was not possible because no control group was included.

This integrated educational approach may be particularly relevant for resource-limited institutions, where access to diverse clinical cases and supervised surgical training may be constrained by ethical, logistical, or patient safety considerations. In such settings, animal model-based simulation offers a feasible and low-cost educational adjunct for teaching surgical anatomy and procedural sequencing, while providing learners with standardized exposure to selected surgical procedures (18, 19). This may support more consistent access to preclinical OMFS training, especially where high-fidelity commercial simulators or human cadaveric specimens are not readily available.

A conceptually similar study demonstrated that simulation-based OMFS training significantly improved learners’ understanding of tooth extraction procedures, procedural confidence, and the translation of theoretical knowledge into clinical practice (20), as demonstrated by Cohort 1’s greater comfort with forceps handling (*P* = 0.033) and preparedness for initial clinical tooth extraction (*P* = 0.028). In contrast, Cohort 2’s preference for textbook-based learning may reflect greater reliance on theoretical resources and was accompanied by a tendency toward lower self-confidence and higher anxiety. These findings are consistent with the significant knowledge gains and retention observed in the present study. However, while study (8) utilized mannequin-based simulation models, the current study employed animal tissue-based models, which may provide additional tactile and anatomical realism through tissue handling, resistance, and procedural response.

The findings reported by Zhong et al. (21) are concordant with prior evidence demonstrating the educational value of animal cadaver models in surgical training. In their study, the use of a pig cadaver model for teaching crown lengthening surgery significantly improved trainees’ procedural understanding and was perceived as highly educational by dental residents. Statistically significant improvements were observed across all assessed knowledge domains (*p* < 0.05), with the exception of determining the appropriate amount of bone removal during osteotomy. Learners rated the overall effectiveness of the model highly, with a mean score of 9.0 out of 10 (21).

Similarly, Devlin et al. demonstrated that cadaveric porcine head models effectively support the teaching of core oral surgical procedures, enabling learners to achieve a better understanding of surgical anatomy and procedural steps compared with conventional teaching alone (18). In their study, the model was perceived very positively by learners, with 96% of students agreeing that the porcine head model was useful for their dental education and expressing willingness to repeat the exercise if given the opportunity (18). These findings indicate that porcine head models represent a practical, low-cost adjunct for teaching fundamental oral surgical procedures.

The educational effectiveness observed in the present study is supported by evidence demonstrating the value of sheep head models in surgical training. Stan et al. reported high satisfaction and effective skill acquisition across trainees with different experience levels when using sheep heads for endoscopic sinus surgery, highlighting their utility for developing manual skills and instrument handling in a realistic yet cost-effective setting (8). These findings align with the current results and reinforce the role of sheep head models as practical educational tools, particularly in developing and resource-limited training institutions.

Within undergraduate and postgraduate OMFS curricula, animal head-based simulation may be positioned as a bridging educational strategy between preclinical instruction and patient-based clinical training (4–6). At the curriculum design level, simulation-based activities enable explicit alignment between learning outcomes, instructional methods, and assessment, thereby supporting outcomes-driven curriculum planning as recommended in contemporary medical education literature (22). During implementation, animal models provide standardized exposure to core surgical concepts and procedures, helping to reduce variation caused by differences in clinical case availability (3).

The selection of sheep head models was based on several anatomical and educational considerations. Sheep heads provide a readily available animal tissue model with oral soft tissues, dentition, mandibular bone, and mucoperiosteal structures that allow trainees to practice key principles of oral surgical access, flap reflection, tissue handling, suturing, bone removal, and graft harvesting (23). Although sheep craniofacial anatomy does not fully replicate human oral and maxillofacial anatomy, the model offers realistic tactile feedback and tissue resistance that can complement conventional classroom-based teaching. The presence of oral mucosa, attached gingiva, teeth, alveolar bone, and neurovascular structures also provides useful opportunities for teaching anatomical orientation, surgical planning, and procedural sequencing in a low-risk environment (24). Therefore, the sheep head model was selected as a practical, low-cost adjunct for preclinical OMFS training, particularly in settings where access to high-fidelity commercial simulators or cadaveric human specimens may be limited.

Although the present study did not objectively assess surgical skill performance, the hands-on nature of the intervention may provide educational advantages over lecture-based teaching alone. Traditional lectures can support conceptual understanding of surgical indications, anatomical landmarks, and procedural steps, but they do not allow learners to practice tactile judgment, instrument handling, tissue manipulation, flap elevation, suturing, or bone removal. In contrast, animal tissue-based simulation provides experiential exposure to tissue resistance, spatial orientation, and procedural sequencing. These features may help learners integrate cognitive knowledge with psychomotor awareness (25). However, because surgical skill was not formally measured in the present study, no conclusion can be made regarding superiority of the intervention over traditional lectures in improving surgical performance. Future controlled studies should include objective skill assessment to determine whether knowledge gains translate into improved procedural competence (26).

## 6 Limitations of the study

As a single-arm study, the absence of a control group limits causal inference. Nevertheless, the use of repeated measures across three time points and rigorous instrument validation strengthens internal validity, and the exploratory design provides effect size estimates to inform future controlled studies.

The study was conducted at a single institution with a moderate sample size, which may limit generalizability to other educational contexts with differing curricula, learner profiles, teaching resources, or clinical exposure. In addition, the use of a per-protocol complete-case analysis may have introduced attrition bias, as 25 of the 76 eligible participants did not complete all assessment time points. Because baseline characteristics of non-completers were not fully available, it was not possible to determine whether non-completers differed systematically from those included in the analysis.

The study focused primarily on knowledge acquisition and short-term retention rather than objective assessment of surgical skill acquisition or clinical performance. Although the intervention provided hands-on exposure to surgical instruments and procedures, including forceps use, scalpel handling, flap design, periosteal elevation, suturing, bone removal, and graft harvesting, these competencies were not assessed using structured observational tools. Future studies should include objective skill assessments, such as procedural checklists, global rating scales, OSCE stations, or video-based blinded assessment, to determine whether knowledge gains translate into procedural competence.

Sheep head models provide useful tactile feedback and allow practice of selected oral surgical procedures. However, they do not fully replicate human oral and maxillofacial anatomy, pathological variability, or the complexity of patient-based clinical care. Therefore, the findings should be interpreted as evidence of improved knowledge and procedural understanding within a simulated training context, rather than direct evidence of transfer to patient care. Learner perceptions were also self-reported and may be subject to response bias, highlighting the need for future studies to include objective performance measures and qualitative data.

Although the knowledge assessment instrument underwent content validity and face validity assessment, its internal consistency reliability was not assessed. Therefore, the reliability of the knowledge scores should be interpreted with caution. In addition, because the same knowledge assessment was administered at T0, T1, and T2 without changing item order, a testing effect cannot be excluded. Future studies should consider using equivalent alternate forms of the assessment or randomizing item order across time points.

## 7 Conclusion

This study demonstrated that integrating small-group case-based learning with hands-on sheep head animal model training resulted in significant and educationally meaningful improvements in OMFS knowledge among undergraduate dental students and interns, with retention maintained at 4 weeks.

The large effect size observed suggests a substantial improvement in knowledge scores following the intervention, while favorable learner perceptions support its acceptability and feasibility. Given its low cost, ethical acceptability, and ability to provide standardized surgical exposure, this approach is particularly well suited to developing and resource-limited educational institutions where patient-based training opportunities may be constrained. Incorporating animal model-based simulation within OMFS curricula may therefore support procedural understanding and preclinical learning; however, future research should focus on comparative controlled designs, objective assessment of procedural skills, and long-term clinical outcomes.

## Data Availability

The raw data supporting the conclusions of this article will be made available by the authors, without undue reservation.

## References

[1] Panagiotidou E Lillis T Fotopoulos I Kalyvas D Dabarakis N. Evaluation of self-perceived confidence and competence in oral surgery among final year undergraduate students in Greece. *Eur J Dent*. (2024) 18:360–7. 10.1055/s-0043-1771330 38158210 PMC10959612

[2] Assael LA. Residency education in oral and maxillofacial surgery: a new curriculum framework. *Oral Maxillofac Surg Clin North Am*. (2022) 34:537–44. 10.1016/j.coms.2022.03.009 36229387 PMC9549297

[3] Swaminathan K T S Haridoss S Thadani M Lakshmi M. Effectiveness of innovative teaching methods versus traditional lectures in dental education: a systematic review and meta−analysis. *J Dent Educ.* (2026). 10.1002/jdd.70160 41631409

[4] Elendu C Amaechi DC Okatta AU Amaechi EC Elendu TC Ezeh CP et al. The impact of simulation-based training in medical education: a review. *Medicine*. (2024) 103:e38813. 10.1097/MD.0000000000038813 38968472 PMC11224887

[5] Seifert LB Schnurr B Herrera-Vizcaino C Begic A Thieringer F Schwarz F et al. 3D-printed patient individualised models vs cadaveric models in an undergraduate oral and maxillofacial surgery curriculum: comparison of student’s perceptions. *Eur J Dent Educ*. (2020) 24:799–806. 10.1111/eje.12522 32133720

[6] Shahrezaei A Sohani M Taherkhani S Zarghami SY. The impact of surgical simulation and training technologies on general surgery education. *BMC Med Educ*. (2024) 24:1297. 10.1186/s12909-024-06299-w 39538209 PMC11558898

[7] Chen CM Lin FH Yang JJ. Simulated spinal durotomy repair for orthopaedic resident training: a perfusion-based porcine cadaveric specimen as an in vitro animal model. *BMC Med Educ*. (2024) 24:1361. 10.1186/s12909-024-06333-x 39587560 PMC11590354

[8] Stan C Ujvary LP Blebea CM Vesa D Tãnase MI Tãnase M et al. Sheep’s head as an anatomic model for basic training in endoscopic sinus surgery. *Medicina*. (2023) 59:1792. 10.3390/medicina59101792 37893511 PMC10608182

[9] Thomas S Jayachandran KP Joseph S Abraham TR. Modelling and simulation in dental education. In: Baiju RM Thomas S editors. *The Dental Teacher.* Cham: Springer Nature Switzerland (2025). p. 85–99.

[10] Nahavandi SR Gholami L Ghojazadeh M Rad AP Tarzemany R. Enhancing oral surgery simulation: a systematic review of 3D-printed patient-specific models compared to traditional animal jaw models for presurgical training. *J Dent Educ*. (2026) 90:433–45. 10.1002/jdd.13992 40696826 PMC13022465

[11] Bergmeister KD Aman M Kramer A Schenck TL Riedl O Daeschler SC et al. Simulating surgical skills in animals: systematic review, costs & acceptance analyses. *Front Vet Sci*. (2020) 7:570852. 10.3389/fvets.2020.570852 33195561 PMC7554573

[12] Faul F Erdfelder E Buchner A Lang AG. Statistical power analyses using G*Power 3.1: tests for correlation and regression analyses. *Behav Res Methods*. (2009) 41:1149–60. 10.3758/BRM.41.4.1149 19897823

[13] Yusoff MSB. ABC of content validation and content validity index calculation. *Educ Med J.* (2019) 11:49–54. 10.21315/eimj2019.11.2.6

[14] Yusoff MSB. ABC of response process validation and face validity index calculation. *Educ Med J.* (2019) 11:55–61. 10.21315/eimj2019.11.3.6

[15] IBM Corp. *IBM SPSS Statistics for Mac OS, Version 31.0.* Armonk, NY: IBM Corp (2025).

[16] Albert FA Seidu AA Mason HM Anderson E Alele FO Heggarty P et al. A systematic review of medical practitioners’ retention and application of basic sciences to clinical practice. *BMC Med Educ*. (2024) 24:997. 10.1186/s12909-024-05952-8 39272053 PMC11396528

[17] Borner U Tschopp S Stewart M Bulut OC Faure F Pabst G et al. The sheep head versus the pig head as a training model for sialendoscopy. *Laryngoscope*. (2025) 135:2741–7. 10.1002/lary.32126 40105215 PMC12255376

[18] Devlin J Ghosh Y Shukla K Forwood M Hurrell M. Utility of cadaveric porcine heads for teaching oral surgical procedures in an Australian dental school: a pilot study. *J Clin Med*. (2024) 13:3032. 10.3390/jcm13113032 38892742 PMC11172896

[19] Salib J Salib M Phillips M. Low-cost simulation kits for surgical training in resource-limited settings: a student-led model. *Cureus*. (2025) 17:e95940. 10.7759/cureus.95940 41341352 PMC12671252

[20] Taysi AE Taysi NM Sismanoglu S. Evaluation of the efficacy of a simulation model used in oral and maxillofacial surgery education. *BMC Med Educ*. (2024) 24:310. 10.1186/s12909-024-05307-3 38504298 PMC10953247

[21] Zhong J Shi D Wang C Zhen M Wei Y Han Z et al. Assessment of a developed pig cadaver model for teaching crown lengthening surgical procedures. *PeerJ*. (2022) 10:e13421. 10.7717/peerj.13421 35669955 PMC9166679

[22] Frank JR Snell LS Cate OT Holmboe ES Carraccio C Swing SR et al. Competency-based medical education: theory to practice. *Med Teach*. (2010) 32:638–45. 10.3109/0142159X.2010.501190 20662574

[23] Al-Qareer AH Afsah MR Müller HP. A sheep cadaver model for demonstration and training periodontal surgical methods. *Eur J Dent Educ*. (2004) 8:78–83. 10.1111/j.1600-0579.2003.00334.x 15059084

[24] Ianacone DC Gnadt BJ Isaacson G. Ex vivo ovine model for head and neck surgical simulation. *Am J Otolaryngol*. (2016) 37:272–8. 10.1016/j.amjoto.2016.01.015 27178523

[25] Bowyer MW Andreatta PB Armstrong JH Remick KN Elster EA. A novel paradigm for surgical skills training and assessment of competency. *JAMA Surg*. (2021) 156:1103–9. 10.1001/jamasurg.2021.4412 34524418 PMC8444063

[26] Dawe SR Pena GN Windsor JA Broeders JA Cregan PC Hewett PJ et al. Systematic review of skills transfer after surgical simulation-based training. *Br J Surg*. (2014) 101:1063–76. 10.1002/bjs.9482 24827930

